# Metabolic crossroads in insulin resistance: exploring lipid dysregulation and inflammation

**DOI:** 10.3389/fimmu.2025.1692742

**Published:** 2025-11-25

**Authors:** Saeede Saadati, Rasoul Godini, Anjana Reddy, Helena Teede, Aya Mousa

**Affiliations:** 1 Monash Centre for Health Research and Implementation (MCHRI), Faculty of Medicine, Nursing and Health Sciences, Monash University, VIC, Melbourne, Australia; 2 Development and Stem Cells Program, Monash Biomedicine Discovery Institute and Department of Anatomy and Developmental Biology, Monash University, Melbourne, VIC, Australia

**Keywords:** insulin resistance, lipid metabolism, chronic inflammation, type 2 diabetes, review

## Abstract

Insulin resistance is a central pathological feature of several chronic metabolic disorders, including obesity, type 2 diabetes, polycystic ovary syndrome, and cardiovascular disease. While its pathogenesis is multifactorial, lipid dysregulation and chronic low-grade inflammation are recognised as two major, interconnected processes that impair insulin action across multiple tissues. This review summarises core mechanistic themes linking these processes, with a focus on three key signalling pathways that are particularly relevant to metabolic regulation and to the interplay between lipid metabolism, inflammation, and insulin action: phosphoinositide 3-kinase/protein kinase B, AMP-activated protein kinase, and c-Jun N-terminal kinase. Dysregulated lipid metabolism, including the accumulation of bioactive intermediates such as diacylglycerols and ceramides, disrupts insulin signalling, promotes lipotoxicity and adipose tissue dysfunction, and triggers inflammatory cascades. In parallel, inflammatory mediators, including cytokines, adipokines, and related signalling pathways, further impair insulin receptor function and exacerbate metabolic stress. Together, these processes form a self-reinforcing cycle that sustains insulin resistance and accelerates disease progression. Despite recent advances in delineating these mechanisms, critical gaps remain in defining tissue-specific effects, pathway interactions, sex-based differences, and the roles of lesser-studied lipid species and regulatory layers, highlighting priorities for future mechanistic research.

## 1 Introduction

Insulin resistance (IR) is highly prevalent, affecting an estimated 16% to 47% of adults worldwide (1). As a central pathophysiological mechanism, IR underlies major chronic metabolic disorders such as type 2 diabetes (T2D), making it a critical target for prevention and treatment. First recognised in the 1930s, IR was initially described in patients who remained hyperglycaemic despite exogenous insulin therapy (2), reflecting the impaired ability of insulin to stimulate glucose uptake and maintain normoglycaemia. It is now recognised as a metabolic state in which insulin-responsive tissues, primary skeletal muscle, adipose tissue, and liver, exhibit reduced responsiveness to insulin action despite normal or elevated circulating insulin levels. This distinguishes IR from conditions of absolute insulin deficiency, such as type 1 diabetes (3, 4). Under normal physiological conditions, insulin secreted from pancreatic β-cells maintains glucose homeostasis by promoting glucose uptake into muscle and adipose tissue, inhibiting hepatic gluconeogenesis, and suppressing lipolysis in adipocytes (3). However, insulin-stimulated glucose uptake in skeletal muscle and adipose tissue is impaired in individuals with IR, while hepatic glucose production remains elevated. To maintain euglycaemia, pancreatic β-cells increase insulin secretion, leading to compensatory hyperinsulinemia. Over time, β-cell function progressively declines, reducing the ability to compensate, leading to worsening hyperglycaemia and glucose intolerance. This is often concomitant with other metabolic sequalae, including elevated free fatty acids, ectopic lipid accumulation, and chronic low-grade inflammation (3, 5, 6).

With systemic consequences extending across multiple organ systems, IR is recognised as a defining feature in a spectrum of chronic disorders. In T2D, sustained IR accelerates β-cell dysfunction, which ultimately results in persistent hyperglycaemia and multisystem complications (7). In gestational diabetes mellitus (GDM), pregnancy-induced IR combined with insufficient β-cell compensation results in maternal hyperglycaemia and adverse perinatal outcomes (8). IR and hyperinsulinemia also contribute to the metabolic and reproductive sequelae of polycystic ovary syndrome (PCOS) by driving or exacerbating androgen excess, disrupting ovarian function and worsening metabolic risk (9). In addition, IR significantly increases the risk of cardiovascular disease (CVD) by promoting endothelial dysfunction, arterial stiffness, and dyslipidaemia (10). Hepatic consequences of IR are seen in metabolic dysfunction-associated steatotic liver disease (MASLD), whereby IR promotes hepatic fat accumulation, liver inflammation, oxidative stress, and fibrosis (11, 12). Obesity compounds each of these conditions by intensifying IR, altering lipid handling, and escalating chronic low-grade inflammation; these effects are influenced by altered body composition especially visceral and ectopic fat accumulation (13). The clustering of these abnormalities - abdominal obesity, dyslipidaemia, dysglycaemia, and hypertension – is captured as metabolic syndrome which is largely driven by IR (14). Beyond metabolic disorders, there is also growing evidence that IR increases the risk of cancer, as hyperinsulinaemia can stimulate tumour growth through mitogenic and anti-apoptotic pathways (15).

Considering its broad clinical implications, understanding the mechanisms that drive and sustain IR is essential. While multiple, interacting pathophysiological processes influence IR, dysregulated lipid metabolism and chronic inflammation are among the most consistently implicated, yet their discrete effects and mechanistic interlinks remain poorly understood. This review outlines key pathways of insulin action and examines how lipid dysregulation, inflammation, and their convergence, influence IR development and severity, with implications for disease progression.

## 2 Key insulin signalling pathways in insulin resistance

Insulin is a 51 amino-acid peptide hormone composed of an α chain (21 amino acids) and a β chain (30 amino acids), linked by two interchain disulphide bonds, with an additional intrachain disulphide bond within the α chain (16). Secreted by pancreatic β-cells, insulin plays a central role in maintaining glucose and lipid homeostasis (16). Binding of insulin to its receptor on target cell membranes triggers a cascade of intracellular signalling events that coordinate key metabolic processes such as glucose uptake, glycogen synthesis, *de novo* lipogenesis, adipogenesis, stimulation of protein synthesis, and suppression of gluconeogenesis and lipolysis (17). However, these signalling pathways are disrupted in IR, leading to metabolic dysfunction and the development of IR-related diseases (17–19). Among the many signalling routes downstream of the insulin receptor, three are particularly relevant to metabolic regulation and to the interplay between lipid metabolism, inflammation, and insulin action, as described below (20–22).

### 2.1 PI3K/Akt pathway in insulin signalling

The phosphoinositide 3-kinase (PI3K)/protein kinase B (Akt) pathway is a key mediator of the metabolic actions of insulin, playing a central role in promoting glucose uptake, glycogen synthesis, *de novo* lipogenesis, adipogenesis, suppressing gluconeogenesis and lipolysis, and stimulating protein synthesis (20). Following insulin binding to its receptor, insulin receptor substrate (IRS)-1 is activated, which in turn recruits and activates PI3K. This leads to the phosphorylation and activation of Akt, a key downstream effector involved in regulating a wide range of metabolic processes (17).

A major function of Akt is the regulation of glucose homeostasis in skeletal muscle, liver, and adipose tissue (2). In skeletal muscle and adipose tissue, Akt promotes glucose uptake by stimulating the translocation of glucose transporter type 4 (GLUT4) to the plasma membrane. GLUT4 trafficking is regulated by Akt via phosphorylation and inhibition of its downstream target, AS160 (Akt substrate of 160 kDa), which allows GLUT4-containing vesicles to fuse with the cell membrane and facilitate glucose entry (2). Akt also stimulates glycogen synthesis in the liver and skeletal muscle by phosphorylating and inactivating glycogen synthase kinase 3, thereby activating glycogen synthase (20, 23), and by activating protein phosphatase 1, which further promotes glycogen synthesis (24). In the liver, Akt suppresses gluconeogenesis by phosphorylating forkhead box protein O1 (FOXO1), a transcription factor that regulates gluconeogenic gene expression (25). Phosphorylation excludes FOXO1 from the nucleus, reducing gluconeogenic enzyme expression and lowering hepatic glucose production (25).

In addition to glucose regulation, Akt plays a central role in lipid metabolism (26). In liver and adipose tissue, Akt stimulates *de novo* lipogenesis, the process by which fatty acids are synthesised from non-lipid sources. This occurs via upregulation of sterol regulatory element-binding protein 1c (SREBP-1c), a transcription factor that activates genes encoding lipogenic enzymes such as acetyl-CoA carboxylase (ACC) and fatty acid synthase (26). In addition to promoting lipid synthesis, Akt inhibits lipolysis by activating phosphodiesterase 3B, which hydrolyses cyclic adenosine 5′-monophosphate (cAMP). The resulting reduction in cAMP levels leads to dephosphorylation of hormone-sensitive lipase (HSL), preventing its translocation to lipid droplets and subsequent hydrolysis of stored triglycerides (3, 27). Together, increased lipogenesis and suppressed lipolysis promote triglyceride storage in liver and adipose tissue, reduce fatty acid oxidation, and support energy balance under normal metabolic conditions (3).

Beyond glucose and lipid regulation, Akt influences protein synthesis and adipogenesis by activating the mechanistic target of rapamycin complex 1 (mTORC1), a central nutrient- and energy- sensing complex that regulates cell growth (28). Here, Akt phosphorylates and inhibits two of its upstream negative regulators: tuberous sclerosis complex 2 (TSC2) and proline-rich Akt substrate 40 (PRAS40) (28), thereby relieving their suppression of mTORC1 activity. In turn, mTORC1 is activated, promoting protein synthesis and adipogenesis, and further contributing to energy storage and tissue growth (28).

Dysregulation of the PI3K/Akt pathway is a key mechanism driving IR and its related metabolic disorders (29). In IR, signalling can be compromised at multiple points, including the insulin receptor, IRS-1, PI3K, Akt, or GLUT4, leading to impaired downstream metabolic effects (3, 29). In skeletal muscle, reduced Akt activation limits GLUT4 translocation, increasing the risk of hyperglycaemia and development of T2D (3). Insufficient Akt signalling in the liver fails to suppress gluconeogenesis, further elevating blood glucose (3). Defective signalling in adipose tissue increases lipolysis and circulating free fatty acids, which exacerbate IR and impair lipid storage and adipogenesis (3). Through these defects, chronic dysregulation of this pathway alters nutrient handling, promotes fat storage and ectopic lipid deposition, reduces fatty acid oxidation, and impairs glucose disposal, collectively driving the development and progression of metabolic disorders including obesity, T2D, MASLD, PCOS, and metabolic syndrome (30–32). The PI3K/Akt pathway is therefore essential for preserving glucose homeostasis and regulating the synthesis of proteins, glycogen, and lipids in a variety of insulin-sensitive tissues.

### 2.2 AMPK pathway in insulin signalling

While insulin primarily activates the PI3K/Akt pathway to promote anabolic processes such as glucose uptake and lipid synthesis, AMP-activated protein kinase (AMPK) is activated under conditions of cellular and/or metabolic stress (e.g., fasting, exercise) when the ratio of adenosine monophosphate (AMP) to adenosine triphosphate (ATP) increases (33–35). Unlike the insulin-stimulated PI3K/Akt pathway, AMPK is largely insulin-independent, although in liver and adipose tissue, insulin may inhibit AMPK due to its opposing catabolic functions (36). AMPK is a heterotrimeric complex composed of a catalytic α-subunit and two regulatory subunits (β and γ) (33). Its activation begins with AMP binding to an allosteric site on the γ-subunit (34), which promotes phosphorylation and protects the enzyme from dephosphorylation, maintaining it in an active state (34). AMPK becomes fully active when upstream kinases, mainly liver kinase B1 and ca2+/calmodulin-dependent kinase kinase 2, phosphorylate Thr172 on its α-subunit (active site) (37, 38). Although liver kinase B1 is constitutively active, protein phosphatases tightly regulate AMPK activity and can deactivate it by dephosphorylating this site. However, AMP binding helps maintain AMPK activity by promoting its phosphorylation and preventing dephosphorylation at the active site (22).

Activated AMPK improves insulin sensitivity and restores metabolic balance through several mechanisms (39). In skeletal muscle, AMPK promotes glucose uptake by phosphorylating AS160; inhibits glycogen synthesis via glycogen synthase-1 phosphorylation; and increases fatty acid oxidation through activation of malonyl-CoA decarboxylase (22, 40). In the liver, AMPK inhibits *de novo* lipogenesis by suppressing SREBP-1c, counteracting the lipogenic effects of insulin (41), and by inhibiting ATP-citrate lyase, an enzyme which converts citrate to cytosolic acetyl-CoA for fatty acid and cholesterol biosynthesis (42). AMPK also inhibits lipid accumulation in liver and muscle by phosphorylating and inactivating ACC, thereby reducing malonyl-CoA levels and promoting mitochondrial fatty acid oxidation (43). These actions collectively decrease hepatic steatosis, suppress gluconeogenesis, and stimulate mitochondrial biogenesis in skeletal muscle, all of which main insulin sensitivity and metabolic efficiency (38).

In addition to its effect on glucose and lipid metabolism, AMPK also suppresses mTORC1 by phosphorylating its upstream regulators, TSC2 and raptor (44, 45). This inhibition counteracts anabolic signalling and reduces excessive protein synthesis, which could otherwise lead to cellular stress and IR. Inhibition of mTORC1 also helps maintain insulin sensitivity by preserving the integrity of insulin receptor substrate proteins (IRS-1 and IRS-2), preventing their degradation and ensuring effective insulin signal transduction (33).

In states of IR, AMPK function can become impaired, and its regulatory control over glucose uptake, lipid metabolism, protein synthesis, and mitochondrial function is compromised, leading to cellular energy imbalance (46). This dysregulation promotes lipid accumulation, and exacerbates inflammation and oxidative stress, which together sustain IR. As such, AMPK acts as a metabolic master switch, and its activation represents a crucial adaptive response to restore cellular homeostasis, mitigate the progression and severity of IR, and ameliorate T2D and its associated complications (39, 46).

### 2.3 JNK-IRS-1 pathway in insulin signalling

The c-Jun N-terminal kinase (JNK) pathway, a member of the mitogen-activated protein kinase family, is a serine/threonine kinase activated in response to different cellular stress stimuli, including inflammatory cytokines, oxidative stress, and elevated free fatty acids (3, 47). Under normal physiological conditions, JNK activity is transient and contributes to adaptive responses by mediating immune responses, cell survival, and apoptosis (3). It regulates transcription factors such as c-Jun (cellular Jun), activating transcription factor 2, and E26 transformation-specific-like protein-1, which together form activator protein-1 complexes that control the expression of genes involved in stress adaptation, inflammation, and cell fate (e.g., survival, proliferation, or apoptosis) (48, 49). In metabolic tissues including the liver, pancreas, adipose tissue, and skeletal muscle, this controlled JNK signalling is essential for maintaining homeostasis, as it modulates immune surveillance, tissue repair, and metabolic flexibility in response to nutrient availability and physiological stressors (50). In healthy conditions, controlled JNK activation therefore supports host defence and tissue integrity. However, when such activation is chronic or dysregulated, it shifts from an adaptive mechanism to a driver of pathological inflammation.

In settings of IR, JNK activation becomes chronic, disrupting insulin signalling and driving metabolic dysfunction (3). A key mechanism by which JNK impairs insulin action is through the phosphorylation of IRS-1 at serine residues, particularly Ser307. This aberrant phosphorylation reduces the ability of IRS-1 to activate downstream signalling via the PI3K/Akt pathway, ultimately leading to impaired glucose uptake, abnormal lipid metabolism, and worsening IR (47, 51). One of the major upstream activators of JNK is angiotensin II, a peptide hormone that underpins the development of diabetic microvascular and macrovascular complications (21, 52). In vascular smooth muscle cells, angiotensin II triggers IRS-1 phosphorylation at serine residues, leading to the degradation of IRS-1 and further impairing its ability to activate downstream signalling pathways such as PI3K/Akt (52). This disruption of insulin signalling worsens hyperglycaemia and promotes dysregulated lipid metabolism by reducing glucose uptake and increasing hepatic glucose production (52).

In addition to insulin signalling defects, chronic JNK activation shifts the metabolic balance toward inflammation and lipid accumulation (53, 54). While JNK does not directly induce lipogenesis via classic transcriptional pathways (e.g., SREBP1), its activation may favour lipid storage in insulin-sensitive tissues by reducing glucose utilisation and modifying energy distribution (53). Concurrently, JNK increases pro-inflammatory signalling by activating nuclear factor-κB (NF-κB), leading to increased expression of inflammatory cytokines. These processes create a vicious cycle in which chronic inflammation and metabolic dysfunction reinforce one another, further exacerbating IR and positioning the JNK–IRS-1 pathway as a central mediator linking cellular stress responses, immune activation, and metabolic dysregulation (54).

Taken together, these disruptions in insulin signalling pathways underscore how cellular stress and metabolic imbalance converge to drive IR. Within this context, inflammation and lipid dysregulation act as overarching drivers that sustain these defects and intensify the metabolic and inflammatory dysfunction characteristic of insulin-resistant conditions.

## 3 Lipid dysregulation and inflammation as drivers of insulin resistance

**Figure 1:**
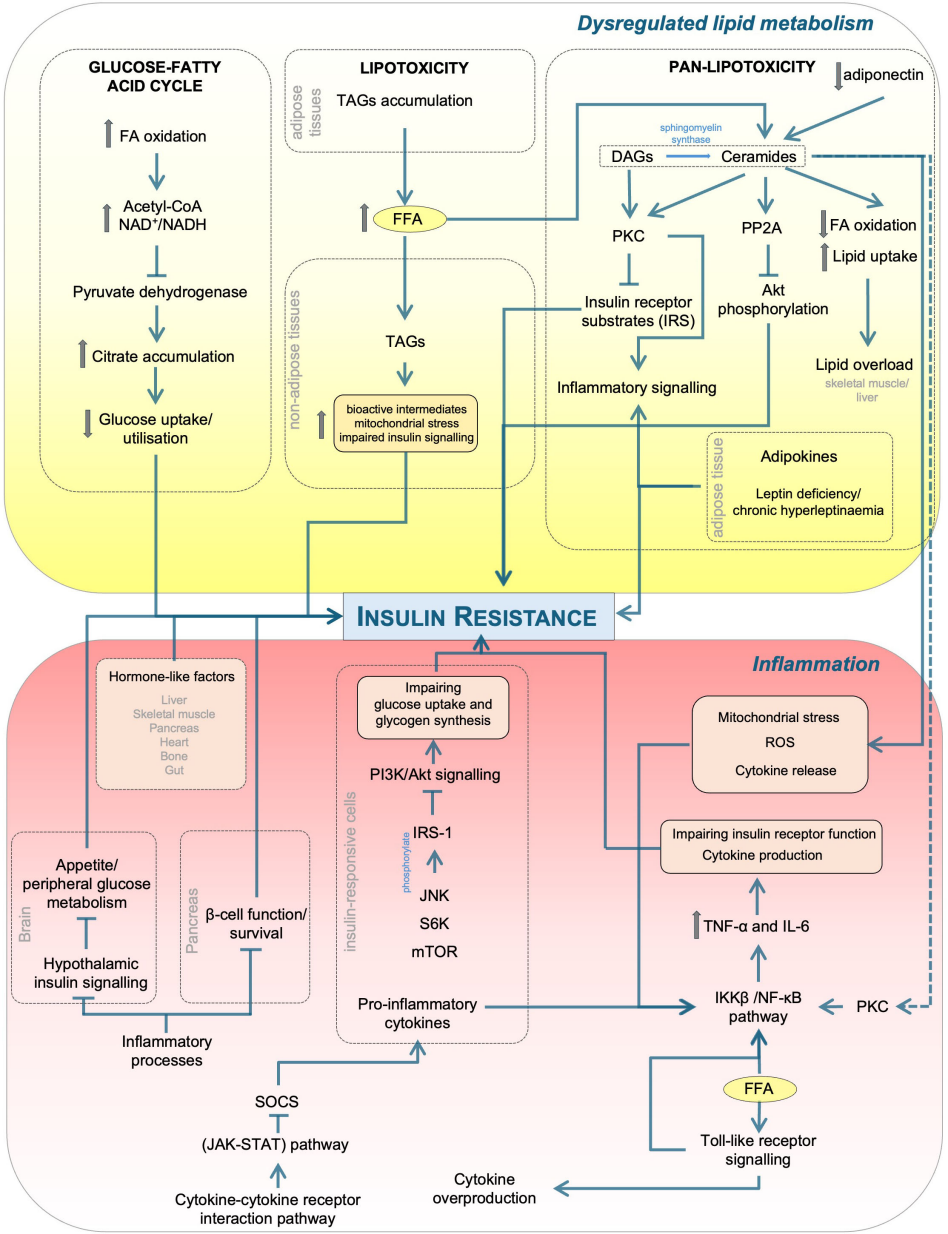
Mechanistic links between lipid dysregulation and inflammation in insulin resistance. Excess FFAs and altered lipid handling generate DAGs and ceramides, activating PKC and PP2A, which inhibit PI3K/Akt signalling and promote lipotoxicity/pan-lipotoxicity. In parallel, TLR activation and cytokines (e.g., TNF-α, IL-6) stimulate IKKβ/NF-κB, JNK, S6K/mTOR, and SOCS/JAK-STAT, driving inhibitory IRS-1 phosphorylation and impaired insulin action across tissues (liver, skeletal muscle, adipose tissue, pancreas, brain). Adipokines (e.g., adiponectin) modulate these effects. Although not depicted here, lipid- or inflammation-induced energy stress can activate AMP-activated protein kinase (AMPK), a key energy sensor that provides a protective counter-regulatory response by mitigating lipotoxic and inflammatory effects. Arrows indicate activation/flow while blunt bars indicate inhibition. PP2A, protein phosphatase 2A; FFA, free fatty acid; FA, fatty acid; ROS, reactive oxygen species; SOCS, suppressor of cytokine signalling; JAK-STAT, Janus kinase-signal transducer and activator of transcription; PKC, protein kinase C; IL, interleukin; TNF-α, tumour necrosis factor-α; IKKβ, IκB kinase β; NF-κB, nuclear factor-κB; JNK, Jun N-terminal kinase; S6K, S6 kinase; mTOR, mechanistic target of rapamycin; IRS-1, insulin receptor substrate 1; PI3K, phosphoinositide 3-kinase; Akt, protein kinase B; NAD^+^/NADH, nicotinamide adenine dinucleotide; TLR, toll-like receptor; DAG/TAG, di/triacylglycerol.

Chronic low-grade inflammation and dysregulated lipid metabolism are well-recognised, interdependent drivers of IR, as illustrated in **Figure 1** (55). Emerging evidence indicates a bidirectional relationship in which excess or altered lipid species promote inflammatory signalling, while inflammation disrupts lipid handling and promotes lipotoxicity. This reciprocal crosstalk is mediated by overlapping molecular pathways involving cytokine signalling, adipokine secretion, and lipid-induced activation of stress and inflammatory kinases, together forming a self-perpetuating cycle that exacerbates IR and metabolic dysfunction.

### 3.1 Lipid dysregulation and insulin resistance

Disturbances in lipid metabolism are a critical and complementary factor in the development of IR (20, 56, 57). One of the earliest models proposed to explain how excess lipids interfere with glucose metabolism is the Randle cycle, also known as the glucose-fatty acid cycle (58, 59). This model suggests that elevated fatty acid oxidation increases mitochondrial by-products, such as acetyl-CoA and nicotinamide adenine dinucleotide, which inhibit pyruvate dehydrogenase activity. The resulting citrate accumulation suppresses key glycolytic enzymes, which results in lowering glucose uptake and utilisation (58, 59). Although foundational, this model does not fully account for the mechanisms underlying lipid-induced IR (60).

In conditions of chronic overnutrition, as the lipid-storage capacity of adipose tissue becomes saturated, excess lipids, primarily in the form of triacylglycerols (TAGs), begin to accumulate in non-adipose tissues such as the liver and skeletal muscle, a phenomenon known as lipotoxicity (61, 62). While TAGs are often considered inert storage molecules, their chronic accumulation reflects sustained lipid oversupply and is closely associated with increased generation of bioactive intermediates, mitochondrial stress, and impaired insulin signalling (63). This ectopic lipid deposition disrupts cellular function caused and is a major factor in IR. Once the storage capacity of adipose tissue is exceeded, it shifts from a passive energy reservoir to an active contributor to metabolic dysfunction, as unregulated lipolysis releases free fatty acids that further exacerbate IR (63).

Traditionally, lipotoxicity referred to the toxic accumulation of triglycerides and free fatty acids in non-adipose tissues (61). This concept has since evolved to include abnormal fat distribution, imbalances in various lipid species, and disruptions in lipoprotein metabolism, all of which lead to more widespread systemic dysfunction (62, 64). Building on this expanded view, the concept of pan-lipotoxicity has emerged (64), extending the definition of lipotoxicity from toxic accumulation of specific lipids within individual tissues to a systemic phenomenon affecting multiple tissues and organs. This systemic toxicity is driven not only by lipid accumulation, but also by widespread disturbances in lipid handling, composition, and transport, including altered levels of ceramides, diacylglycerols (DAG), and lipoproteins (64, 65), which collectively promote cellular damage and dysfunction. This broader framework better captures the complexity of conditions underpinned by IR, facilitating a more holistic understanding of the lipid metabolism pathways involved in IR-related disorders (64).

Recent research has highlighted bioactive lipid intermediates, such as DAGs and ceramides, as important mediators of impaired insulin signalling (20). These molecules inhibit phosphorylation and activation of insulin receptor substrates by activating specific isoforms of protein kinase C (PKC), such as PKC-θ in muscle and PKC-ϵ in the liver (66–68). Experimental inhibition or genetic deletion of these PKC isoforms improves insulin sensitivity, supporting a causal link (69, 70). However, the role of DAGs in IR remains contentious, as some studies show that deletion of PKC-ϵ in the liver does not consistently improve insulin sensitivity, and insulin receptor phosphorylation at the implicated sites is often undetectable (71). These discrepancies highlight the need for further research to determine the exact role of the DAG-PKC axis in lipid-induced IR.

Ceramides, a class of sphingolipids, are also important lipid intermediates that hinder insulin signalling through several mechanisms (72). Ceramides activate protein phosphatase 2A (PP2A), inhibiting Akt phosphorylation, a key step in the insulin signalling cascade (73). They also suppress fatty acid oxidation and enhance lipid uptake, increasing lipid overload in skeletal muscle and liver (72). Insulin-resistant tissues have high concentrations of ceramides, particularly C16 and C18 species synthesised by ceramide synthase 6 (74, 75). Ceramide accumulation is further driven by elevated circulating free fatty acids and reduced adiponectin, both common features of IR, which respectively increase ceramide synthesis and impair its clearance by downregulating ceramidase activity (76, 77). Adiponectin connects lipid metabolism to adipokine signalling by stimulating ceramidase activity via its receptors AdipoR1 and AdipoR2, in turn promoting ceramide degradation (78). In addition to directly impairing insulin signalling, ceramides exacerbate IR by inducing mitochondrial stress, generating reactive oxygen species, and promoting pro-inflammatory cytokine release (3). DAGs and ceramides are biochemically interconnected, as sphingomyelin synthase converts ceramides into DAGs (3). Both lipids can activate PKC isoforms, which phosphorylate Raf kinase inhibitory protein (RKIP), leading to its dissociation from Raf and subsequent activation of the mitogen-activated protein kinase/extracellular signal-regulated kinase (MEK-ERK) signalling cascade (79, 80). Activation of this pathway enhances NF-κB activity and promotes the transcription of pro-inflammatory cytokines, thereby linking lipid-induced PKC activation to inflammation and worsening IR (80). Excessive lipid supply, particularly of free fatty acids, can also activate pattern recognition receptors such as Toll-like receptor 4, further amplifying cytokine production and worsening IR (3).

Overall, disturbances in lipid metabolism contribute to IR through both well-established and newly recognised mechanisms. Although the Randle cycle explained some of the early effects of lipid oversupply on glucose metabolism, additional pathways have since been identified that extend and complement this framework in the context of IR. Expanded concepts of lipotoxicity and pan-lipotoxicity emphasise that IR arises not only from localised lipid excess, but from systemic disturbances in lipid storage, distribution and trafficking, compounded by bioactive intermediates such as DAGs and ceramides. These processes impair insulin signalling and promote metabolic stress and inflammation, emphasising the need for therapeutic approaches that target systemic lipid dysregulation as a central driver of IR.

### 3.2 Inflammation and insulin resistance

Chronic low-grade inflammation reflects an imbalance between pro-inflammatory cytokines, such as interleukin (IL)-6, IL-1β, and tumour necrosis factor-α (TNF-α), and anti-inflammatory mediators such as IL-10 and transforming growth factor-β1 (TGF-β1) (81). Elevated pro-inflammatory cytokines, produced primarily by activated immune cells and/or adipose tissue in the context of IR, induce paracrine activation of nearby serine kinases in insulin-responsive cells (e.g., adipocytes, hepatocytes), including IκB kinase β (IKKβ), JNK, S6 kinase (S6K), and mTOR (17, 82). These kinases phosphorylate IRS-1 on serine residues, disrupting downstream PI3K/Akt signalling and impairing glucose uptake and glycogen synthesis in target tissues such as liver and muscle (17). Inflammatory processes can also impair insulin action in the pancreas by reducing β-cell function and survival; and in the brain by disrupting hypothalamic insulin signalling that regulates appetite and peripheral glucose metabolism (83). Together, these mechanisms perpetuate systemic IR via a bidirectional loop, where inflammation reduces insulin responsiveness, and this diminished insulin action potentially exacerbates weight gain, amplifying inflammatory cytokine production and driving progressive adiposity, metabolic dysfunction and T2D risk (83, 84).

Pro-inflammatory effects on insulin signalling are mediated via several key interconnected pathways, including the NF-κB pathway, Toll-like receptor signalling, and cytokine-cytokine receptor interactions (85, 86). NF-κB is activated by metabolic stressors such as elevated circulating free fatty acids (especially palmitate), and indirectly by bioactive lipids such as DAGs and ceramides, through upstream kinases, oxidative stress and receptor-mediated pathways. This in turn induces transcription of inflammatory genes such as TNF-α and IL-6 that impair insulin receptor function (85, 86). IKKβ, an upstream regulator in this pathway, activates NF-κB by phosphorylating and degrading its inhibitor, IκB, allowing NF-κB to translocate to the nucleus and initiate the expression of pro-inflammatory genes (87). This pathway promotes cytokine production in adipocytes, hepatocytes, and macrophages, particularly in the context of obesity or high-fat diet conditions (86). Both the JNK and IKKβ/NF-κB pathways converge on IRS-1, leading to its inhibitory serine phosphorylation, which impairs insulin signal transduction and contributes to systemic IR and associated metabolic dysfunction (86). In parallel, Toll-like receptor signalling enhances NF-κB activation and cytokine overproduction, especially through toll-like receptor 4 and the adaptor protein MyD88. This pathway is activated in part by saturated free fatty acids, a key mechanistic link between lipid oversupply and inflammatory signalling in IR (85). Further, the cytokine-cytokine receptor interaction pathway augments immune cell activation and signalling and, through activation of the Janus kinase-signal transducer and activator of transcription (JAK-STAT) pathway, induces suppressor of cytokine signalling (SOCS) protein expression, further reinforcing this pro-inflammatory state (85). Together, these pathways impair insulin signalling by inducing inhibitory serine phosphorylation of IRS-1 and activating downstream kinases that further disrupt the PI3K/Akt signal transduction, thereby contributing to systemic IR (85).

In addition to systemic inflammatory cytokine signalling, adipose tissue itself plays a pivotal role in modulating insulin sensitivity, by secreting adipokines and other bioactive compounds with potent immunometabolic effects (88). Chronic unresolved inflammation, both in systemic circulation and within adipose depots, drives the pathogenesis of obesity-related cardiometabolic disease (89). Leptin, the first adipokine identified, regulates energy balance and inflammatory responses through signalling via the long isoform of its receptor (LEPRB) in the brain and immune cells (88, 90). Both leptin deficiency and chronic hyperleptinaemia promote IR; leptin deficiency impairs energy balance and glucose regulation, while excess leptin, which is common in obesity, causes leptin resistance and blunts its metabolic effects (91, 92). In contrast, adiponectin is an anti-inflammatory, anti-diabetic adipokine that lowers lipotoxicity, in part by enhancing ceramide clearance through adiponectin receptor-mediated ceramidase activity, thereby enhancing insulin sensitivity and protecting against tissue inflammation (88, 93). It also promotes fatty acid oxidation, preserves pancreatic β-cell function, and exerts anti-apoptotic and anti-fibrotic effects (3). Higher serum adiponectin levels have been associated with a lower risk of metabolic and obesity-related disorders, such as T2D and GDM (94, 95). Similar to adiponectin, apelin also improves insulin sensitivity by increasing Akt phosphorylation and glucose uptake via the AMPK pathway (96), with studies reporting higher apelin concentrations in insulin-resistant and/or individuals with morbid obesity and T2D (88, 97). Other adipokines such as resistin and chemerin have also been implicated in IR. Resistin promotes IR primarily through increasing inflammation in adipose tissue and other metabolic organs, and may indirectly contribute to lipotoxicity via macrophage activation, although a direct causal link in humans remains unclear (98, 99). In contrast, chemerin has context-specific effects that depend on tissue type and metabolic state. At physiological levels, it may modulate insulin sensitivity through regulation of IRS-1, GLUT4, and adiponectin expression, particularly in adipose tissue and skeletal muscle. However, when chronically elevated, as observed in obesity and metabolic inflammation, chemerin can contribute to lipotoxicity by impairing fatty acid clearance and promoting hepatocyte lipid accumulation, potentially worsening IR. These roles remain controversial and are likely dependent on local tissue environment, the form of chemerin present and stage of metabolic disease (100–103).

In addition to cytokines and adipokines, hormone-like factors released by the liver (hepatokines such as fetuin-A and fibroblast growth factor-21), skeletal muscle (myokines such as irisin), pancreas (e.g., amylin), heart (cardiokines such as B-type natriuretic peptide), bone (osteokines such as osteocalcin), and gut (enterokines/incretins such as glucagon-like peptide-1 and peptide YY) also influence insulin sensitivity and energy metabolism, highlighting the complex multi-organ crosstalk involved in metabolic regulation (104, 105). Many of these interconnected signals are modulated by inflammatory pathways, providing a mechanistic link by which chronic inflammation can disrupt insulin signalling and contribute to the development of IR (104).

Collectively, available evidence underscores the importance of inflammation as a central driver of IR, sustained by the interplay between classical pro-inflammatory cytokines, adipose-derived and hormone-like mediators, and bioactive lipid species. Adipose tissue in particular functions as a dynamic immunometabolic organ, rather than a passive energy reservoir, integrating systemic inflammatory cues with local adipokine and lipid secretion to influence insulin sensitivity across multiple tissues. In this context, lipid intermediates such as ceramides and DAGs can trigger inflammatory signalling, while inflammatory cytokines and altered adipokine profiles disrupt lipid storage, oxidation, and trafficking, thereby perpetuating lipotoxicity. Protective adipokines such as adiponectin and apelin exhibit insulin-sensitising effects, whereas dysregulation of other mediators such as leptin, resistin, and chemerin, together with hormone-like signals (e.g., fetuin-A, irisin, etc.) converge with lipid-induced inflammatory signalling to exacerbate metabolic dysfunction. Targeting these interconnected lipid-inflammation networks, spanning cytokine signalling, adipose tissue function and broader multi-organ cross-talk within the pan-lipotoxicity framework, offers a promising strategy to restore metabolic homeostasis and reduce the burden of metabolic disorders including T2D.

## 4 Limitations and future directions

Despite substantial progress in elucidating the molecular mechanisms linking lipid dysregulation, inflammation and IR, several key limitations and knowledge gaps remain. First, much of the current evidence is derived from animal models and *in vitro* experiments which, while invaluable for generating mechanistic insights under controlled conditions, do not directly and fully reflect the complexity of human IR. These models often oversimplify biological systems and are constrained by species-specific differences in immune responses, hormonal regulation, and metabolic processes. For example, metabolic profiles and responses to interventions in rodent models, commonly used in IR research, differ markedly from those in humans, especially in relation to sex hormone influences, depot-specific adipose tissue function, and the regulation of inflammatory pathways (106, 107). *In vitro* studies similarly tend to isolate specific cell types or pathways, thereby overlooking the complex crosstalk and systemic interactions that occur *in vivo*. Such differences have contributed to translational failures; for example, AMPK activators that improved lipid metabolism and IR in animal models have failed to replicate the same beneficial effects in human trials (38). Consequently, findings from these models may overestimate or underestimate the clinical relevance of specific molecular pathways and their potential as therapeutic targets in diverse human populations. To complement insights from existing animal and *in vitro* studies, future research should incorporate physiologically relevant human-based models, such as advanced 3D culture systems, organoids, and micro-physiological platforms, xenografts using human adipose or hepatic tissues, alongside *ex vivo* human tissue studies, prospective cohorts with deep phenotyping, and multi-omics approaches to capture the complex, multi-layered regulation of metabolic and inflammatory networks in IR.

Second, pathway-specific and study-related factors limit our understanding of how insulin signalling networks interact under various metabolic conditions. Although the PI3K/Akt and AMPK pathways are well-established in regulating glucose and lipid metabolism, their dynamic crosstalk in complex clinical settings such as obesity, T2D, GDM, and PCOS remains poorly defined, including whether they act in compensatory, synergistic, or antagonistic ways. This uncertainty is further complicated by inconsistencies across studies, including differences in disease models, tissue-specific responses, and control for inflammatory states. For example, while AMPK is activated by cellular energy stress and PI3K/Akt is typically insulin-driven, it is unclear how these processes influence each other when active in the same metabolic environment, or how their combined activity amplifies, counteracts, or modifies downstream effects on inflammation, insulin sensitivity, and tissue-specific metabolic functions. The JNK pathway, which is frequently linked to IR through IRS-1 inhibition and inflammatory signalling, is similarly complicated by variations in its upstream activators and downstream effects across tissues and metabolic settings. These variations can obscure the role of JNK in systemic IR, particularly in the context of lipid overload and chronic low-grade inflammation. Together, these issues make it challenging to pinpoint which pathways should be targeted therapeutically, and in whom. Addressing these gaps will require coordinated, cross-tissue and longitudinal human studies supported by integrative analytical approaches to map the temporal and spatial interactions of these pathways and identify context-specific therapeutic targets.

Another emerging challenge is the growing identification of novel regulators in IR pathogenesis, such as non-coding RNAs, epigenetic modifications, and gut microbiota-derived metabolites. These factors remain largely absent from standard animal and cell-based models used to study IR, despite mounting evidence from preclinical and human studies linking them to the modulation of insulin signalling (108, 109). This gap reflects both the complexity of these regulatory networks and the limited research examining how they interact with classical insulin signalling pathways such as PI3K/Akt and AMPK or stress-activated pathways such as JNK. Efforts to assess the clinical significance and therapeutic potential of these novel regulators are further constrained by variations in study designs, population characteristics, and analytical techniques. Progress will require incorporating these novel regulatory layers into established mechanistic frameworks, particularly in well-characterised human cohorts, and applying multi-omics and systems biology approaches to determine their role in disease heterogeneity and to uncover novel, context-specific therapeutic targets.

Beyond these emerging regulatory factors, important knowledge gaps also remain for other bioactive lipid intermediates in the context of IR. While DAGs and ceramides are well-established as lipid intermediates contributing to IR, other species including acylcarnitines, lysophospholipids, sphingomyelins, and free fatty acids, likely play important but underexplored roles. Branched fatty acid esters of hydroxy fatty acids (FAHFAs) enhance insulin sensitivity and reduce inflammation, yet their circulating levels are reduced in IR (3, 89) and their roles in complex metabolic disorders such as T2D and GDM are not well understood. The contribution of these diverse lipids to the broader “pan-lipotoxicity” framework, and their interactions with cytokine and adipokine networks, remain poorly defined. Expanding lipidomics beyond conventional targets, using both targeted and untargeted approaches, will be critical for mapping these lipid-inflammation networks and clarifying the functional significance of lesser-studied lipid species in IR pathogenesis.

Finally, research on sex-specific variations in insulin signalling and IR remain underrepresented in the literature. Many clinical studies do not stratify analyses by sex, and most preclinical studies use male animals to avoid variability brought about by hormonal fluctuations. Yet, evidence indicates that sex hormones can markedly influence insulin sensitivity, adipose tissue distribution, and inflammatory responses (110–114). Common conditions such as PCOS provide a clear example of this, with androgen excess in women contributing to IR via alterations in adipose tissue biology, lipid handling, and inflammatory signalling (115). Recognising such differences is essential, as sex can shape both the mechanisms and severity of IR, with direct consequences for prevention and treatment. Future research should routinely incorporate sex as a biological variable, prioritise mechanistic and clinical studies in women, and apply sex-stratified analyses to inform targeted prevention and treatment strategies.

## 5 Conclusions

IR is a complex metabolic disorder primarily driven by the disruption of key intracellular signalling pathways, including PI3K/Akt, AMPK, and JNK. These pathways regulate essential cellular processes underpinning IR, including glucose uptake, glycogen synthesis, lipid metabolism and inflammatory responses. Impaired Akt activation, decreased AMPK activity, and increased JNK signalling decrease insulin sensitivity and promote lipid accumulation, chronic low-grade inflammation, and overall metabolic dysfunction. The interplay between these defects creates a self-reinforcing cycle that exacerbates IR and accelerates disease progression. Despite significant advances in our understanding of the molecular drivers of IR, critical gaps remain in defining tissue-specific differences in signalling, pathway crosstalk under metabolic stress, the roles of novel regulators and diverse lipid intermediates, and sex-specific mechanisms. Addressing these challenges requires leveraging physiologically relevant human models, integration of metabolic and inflammatory networks, and multi-omics approaches in well-characterised cohorts. Such strategies will be essential for identifying context-specific therapeutic targets and developing precise, effective treatments for IR and its associated metabolic disorders.
